# Reversible Conduction Failure in Anti-lactosylceramide-antibody-positive Combined Central and Peripheral Demyelination

**DOI:** 10.3389/fneur.2019.00600

**Published:** 2019-06-07

**Authors:** Masaya Harada, Shiroh Miura, Hiroshi Kida, Taiga Moritaka, Ken-ichi Irie, Takashi Kamada, Yusuke Uchiyama, Sayuri Shima, Tatsuro Mutoh, Tomoaki Hoshino, Takayuki Taniwaki

**Affiliations:** ^1^Division of Respirology, Neurology and Rheumatology, Department of Medicine, Kurume University School of Medicine, Kurume, Japan; ^2^Department of Radiology, Kurume University School of Medicine, Kurume, Japan; ^3^Department of Neurology, Fujita Health University School of Medicine, Aichi, Japan

**Keywords:** combined central and peripheral demyelination (CCPD), encephalomyeloradiculoneuropathy (EMRN), lactosylceramide, reversible conduction failure (RCF), total plasma exchange, steroid, therapy

## Abstract

We describe a 60-year-old woman with combined central and peripheral demyelination who presented with obstinate constipation, weakness in the lower limbs, and a bilateral sensory disturbance below her chest followed by girdle sensation in the right region of the abdomen, which was responsive to steroid therapy and plasmapheresis. Serum anti-lactosylceramide antibody was positive without anti-neurofascin 155 antibody or anti-galactocerebroside antibody positivity. Two months later, the patient had a first relapse that was responsive to steroid treatment. A nerve conduction study confirmed reversible conduction failure (RCF) in both episodes. Our case is unique in that she had an RCF episode as well as some similarities to encephalomyeloradiculoneuropathy.

## Background

Combined central and peripheral demyelination (CCPD) is characterized by demyelination in the central and peripheral nervous systems (1, 2). Recent studies revealed the following CCPD-associated autoantibodies: anti-neurofascin155 antibody, anti-neurofascin186 antibody, anti-galactocerebroside antibody, and anti-lactosylceramide antibody (1–4).

Lactosylceramide is a glycosphingolipid that is thought to be expressed on glial cells and neurons in the central nervous system (CNS) and peripheral nervous system (5–7). Anti-lactosylceramide antibody, which is positive in most encephalomyeloradiculoneuropathy (EMRN) patients, is thought to be related to inflammation in the CNS (4, 6, 8, 9). CCPD and EMRN both exhibit CNS and peripheral nervous system impairments. However, CCPD is characterized by multifocal acquired inflammatory demyelinating sensory and motor neuropathy followed by CNS impairments. The clinical features of CCPD include a chronic onset and a relapsing-remitting course, albumin-cytologic dissociation of cerebrospinal fluids, and a low frequency of oligoclonal IgG bands (OCB) positivity, fulfilling the European Federation of Neurological Societies criteria for definite chronic inflammatory demyelinating polyradiculoneuropathy (4, 10). In contrast, EMRN is an acute or subacute progressive disease that causes encephalitis, myelitis, radiculitis, and peripheral neuritis. In EMRN, peripheral neuropathy is axonal, with or without demyelinating neuropathy (5, 10). EMRN patients often have subacute motor weakness, decreased consciousness, and autonomic dysfunction (4).

In this report, we present a 60-year-old woman with serum anti-lactosylceramide-antibody-positive CCPD who showed reversible conduction failure (RCF) following steroid therapy and plasmapheresis. Our case is similar to EMRN. We also discuss the pathogenesis of this case based on responsiveness to treatment.

## Case Presentation

A 60-year-old woman had noted obstinate constipation. One month later, she developed weakness in the lower limbs and bilateral paresthesia below the chest. One month later, she was admitted to our hospital because of a girdle sensation in the right region of the abdomen and progressive severe weakness in the lower limbs. She showed sensory impairment of all modalities below Th6 dermatomes on the right and below Th8 dermatomes on the left. Deep tendon reflexes were absent in the right upper and lower limbs. Bilateral Babinski reflexes were observed, and cranial nerve examination identified no abnormalities. There was no bladder dysfunction.

**Figure 1:**
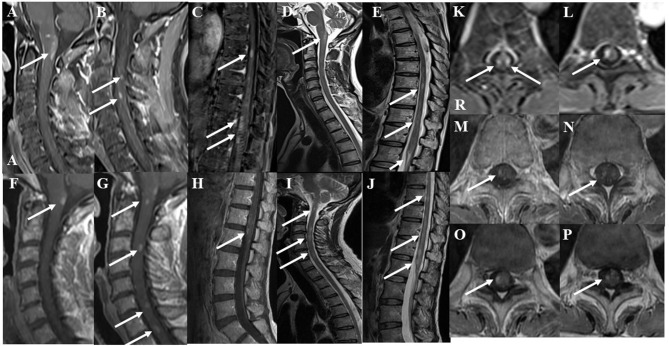
Gadolinium-enhanced spinal magnetic resonance imaging (MRI) findings at the initial attack. The lower “A” represents “anterior” in **(A)**. The “R” represents “right” in **(K)**. **(A–C)** Post-contrast fat-suppression T1-weighted images showed multiple abnormal foci in the medulla and spinal cord at the initial attack. **(D,E)** T2-weighted images showed multiple abnormal foci in the medulla and spinal cord at the initial attack. **(F–H)** Post-contrast T1-weighted MRI of the cervicothoracic and lumbar spine after two courses of high-dose steroid pulse therapy. Abnormal enhancement was slightly decreased, but new enhanced lesions appeared in the spinal cord at the 3rd, 6th, and 7th levels of the cervical spine. **(I,J)** T2-weighted MRI of the cervicothoracic and lumbar spine after two courses of high-dose steroid pulse therapy. T2-weighted images showed patchy lesions with a marked increase in signals in the spinal cord at the 3rd and 6th levels of the cervical spine and at the 9, 10, and 12th levels of the thoracic spine. **(K,L)** Axial fat-suppression T1-weighted images showed enhanced lesions at the 9 and 12th levels of the thoracic spine at the initial attack. **(M,N)** After two courses of high-dose steroid pulse therapy, follow-up T1-weighted MRI showed that abnormal enhancement was decreased. **(O,P)** After three courses of high-dose steroid pulse therapy and 5 plasmapheresis treatments, follow-up MRI showed that the enhanced foci were diminished but still present.

Serum anti-aquaporin 4, anti-myelin oligodendrocyte glycoprotein, anti-glycolipid (GM1, GM2, GM3, GD1a, GD1b, GD3, GT1b, GQ1b, galactocerebroside), and anti-neurofascin155 antibodies were all negative. Serum anti-lactosylceramide antibody (IgG) was positive, while serum anti-glucoceramide antibody and cerebrospinal fluid (CSF) anti-lactosylceramide antibody (IgG) were equivocal. CSF analysis revealed pleocytosis (35 cells/μL, 100% mononuclear) and an elevation of total protein (83 mg/dL). Myelin basic protein level was high (818 pg/mL) and the IgG index was upregulated (1.54). OCB were positive. Gadolinium-enhanced magnetic resonance imaging (MRI) demonstrated multiple foci of abnormal signal intensities in the medulla and spinal cord (Figure 1). Neither ovoid lesions nor Dawson's fingers were observed.

**Figure 2:**
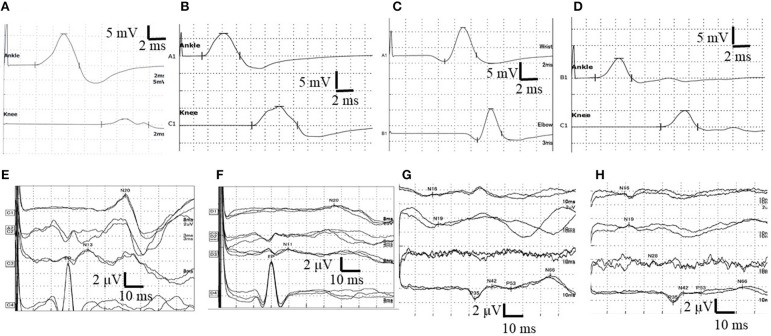
Nerve conduction study (NCS) in the right tibial nerve and the results of right side short-latency somatosensory evoked potentials (SSEPs). **(A)** The waveform of NCS findings at first admission. Conduction block was observed. **(B)** The waveform of NCS findings at 11 days after first admission. Normal NCS. **(C)** The waveform of NCS findings at the second admission. NCS revealed prolonged distal motor latency (DML) and decreased motor nerve conduction velocity (MCV). **(D)** The waveform of NCS at 14 days after second admission. Normal NCS. **(E)** The waveform of SSEPs in the right upper limb before treatment at the initial attack. Normal SSEPs. **(F)** The waveform of SSEPs in the right upper limb after two courses of high-dose steroid pulse therapy and four selective plasma exchanges at the initial attack. N13 and N20 were not evoked. **(G)** The waveform of SSEPs in the right lower limb before treatment at the initial attack. Normal SSEPs. **(H)** The waveform of SSEPs in the right lower limb after two courses of high-dose steroid pulse therapy and four selective plasma exchanges at the first attack. Central conduction time (N19–P35) was normal but prolonged from 20.4 (in the first SSEPs) to 23.9 ms (normal values: 24.2–25.2 ms). N13: negative peak recorded from the posterior column at the C7 level of the spinal cord ~13 ms after median nerve stimulation. N20: negative peak recorded from the contralateral scalp ~20 ms after median nerve stimulation. N19: negative peak recorded from the posterior column at the Th12 level of the spinal cord ~19 ms after peroneal nerve stimulation. P35: positive peak recorded from the cortex ~35 ms after peroneal nerve stimulation. N19–N35: inter-peak latency, central sensory conduction time.

A nerve conduction study (NCS) revealed reduced compound muscle action potential (CMAP) amplitudes in the bilateral peroneal nerves (right and left: 0.8 and 0.9 mV, respectively). Conduction block was observed in the right tibial nerve (distal and proximal CMAPs: 10.7 and 1.9 mV, respectively; Figure 2). Distal motor latency (DML) was prolonged in the right peroneal nerve (6.0 ms). A sensory nerve action potential was not evoked in the left sural nerve. In addition, the F wave was absent in the bilateral peroneal nerves (Table 1). The peripheral nerve involvement was asymmetric and resembled multifocal acquired demyelinating sensory and motor (MADSAM) neuropathy, which is a subtype of chronic inflammatory demyelinating polyradiculoneuropathy. Short-latency somatosensory evoked potentials (SSEPs) and visual evoked potentials were normal (Table 2, Figure 2).

**Table 1:** Nerve conduction studies on both sides at the initial attack, before treatment.

	**Normal value**	**Left side**	**Right side**
Age at examination		60	
**Median nerve**			
MCV (m/s)	>47	60.4	ND
Distal CMAP (mV)	>4	14.4	ND
Proximal CMAP (mV)		12.0	ND
DML (ms)	<4.5	3.0	ND
F wave occurrence (%)	>70	81.0	ND
Minimum F wave latency (ms)	<31	26.3	ND
SCV (m/s)	>46	49.2	ND
SNAP (μV)	>9	29.1	ND
**Ulnar nerve**			
MCV (m/s)	>47	67.6	ND
Distal CMAP (mV)	>3	9.2	ND
Proximal CMAP (mV)		5.4	ND
DML (ms)	<4	2.6	ND
F wave occurrence (%)	>70	**43.0**	ND
Minimum F wave latency (ms)	<27	25.5	ND
SCV (m/s)	>46	52.8	ND
SNAP (μV)	>9	62.5	ND
**Tibial Nerve**			
MCV (m/s)	>39	40.8	**40.0**			
Distal CMAP (mV)	>3	10.9	10.67
Proximal CMAP (mV)		9.0	**1.90**
DML (ms)	<6.3	4.0	3.8
F wave occurrence (%)	>70	93.0	**31.0**
Minimum F wave latency (ms)	<58	51.3 (A wave)	52.5
**Peroneal nerve**			
MCV (m/s)	>40	46.8	41.0
Distal CMAP (mV)	>2.5	**0.9**	**0.8**
Proximal CMAP (mV)		**0.6**	**0.6**
DML (ms)	<5.5	4.6	**6.0**
F wave occurrence (%)	>70	**0.0**	**0.00**
Minimum F wave latency (ms)	<56	**Not evoked**	**Not evoked**
**Sural nerve**			
SCV (m/s)	>39	**Not evoked**	45.5
SNAP (μV)	>4	**Not evoked**	12.2

*MCV, motor conduction velocity; CMAP, compound muscle action potential; DML, distal motor latency; SCV, sensory conduction velocity; SNAP, sensory nerve action potential; ND, not done. Abnormal values are expressed in bold*.

**Table 2:** Short latency somatosensory evoked potentials at the initial attack, before treatment, and after three courses of high-dose steroid pulse therapy and three plasma exchange treatment.

	**Before treatment**	**After treatment**	**Normal data**
**RIGHT UPPER LIMB**			
EP	8.9	9.0	8.97 ± 0.55
N11		**11.8**	10.84 ± 0.47
N13	12.1	**Not evoked**	12.54 ± 0.54
N20	18.4	**19.7**	18.39 ± 0.91
N13–N20	6.3	**Not evoked**	5.9 ± 0.4
**RIGHT LOWER LIMB**			
N16	**19.7**	**19.5**	<19.0
N19	**22.9**	21.9	<22.0
N28	**Not evoked**	**34.9**	<33.0
P35	43.3	**45.8**	<44.0
N42	52.9	51.4	ND
P53	63.6	61.0	ND
N66	85.4	83.4	ND
N19–P35	20.4	23.9	<28.3

Median nerve stimulation.

EP: negative peak recorded from Erb's point ~9 ms after the stimulus.

N11: negative peak recorded ~11 ms after the stimulus.

N13: negative peak recorded from the posterior column at the C7 level of the spinal cord ~13 ms after the stimulus.

N20: negative peak recorded from the contralateral scalp ~20 ms after the stimulus.

N13—N20: inter-peak latency, central sensory conduction time.

Peroneal nerve stimulation.

N16: negative peak recorded from the posterior column at the L4 level of the spinal cord ~16 ms after the stimulus.

N19: negative peak recorded from the posterior column at the Th12 level of the spinal cord ~19 ms after the stimulus.

N28: negative peak recorded at ~28 ms after the stimulus.

P35: positive peak recorded from the cortex (Cz') ~35 ms after the stimulus.

N42: negative peak recorded from the cortex ~42 ms after the stimulus.

P53: positive peak recorded from the cortex ~53 ms after the stimulus.

N66: negaitive peak recorded from the cortex ~66 ms after the stimulus.

*N19–N35: inter-peak latency, central sensory conduction time*.

Abnormal values are expressed in bold.

Upon treatment with methylprednisolone pulse therapy (1,000 mg/day for 3 days, two times), RCF was observed in NCS (Table 3, Figure 2); however, the patient's muscle strength in the right upper limb was weakened on day 8 post-admission. Post-contrast-enhanced spinal MRI demonstrated abnormal enhanced lesions at the C3 to C4 level (Figure 1). SSEPs revealed that the right central conduction time (N19–P35) was normal, but prolonged from 20.4 (in the first SSEP) to 23.9 ms (normal values: < 28.3 ms), and right N13 and N20 negative peaks were not observed (Table 2, Figure 2). Selective plasma exchange (1.25 plasma volume exchange with 5% albumin replacement fluid; selective plasma exchange procedures were performed twice a week for 2 weeks) and additional methylprednisolone pulse therapy only improved muscle strength in the right upper limb.

**Table 3:** Nerve conduction studies on both sides after two courses of high-dose steroid pulse therapy at the initial attack.

	**Normal value**	**Left side**	**Right side**
Age at examination		60	
**Median nerve**			
MCV (m/s)	>47	ND	52.1
Distal CMAP (mV)	>4	ND	4.7
Proximal CMAP (mV)		ND	4.6
DML (ms)	<4.5	ND	3.0
F wave occurrence (%)	>70	ND	81.0
Minimum F wave latency (ms)	<31	ND	24.6
SCV (m/s)	>46	ND	57.8
SNAP (μV)	>9	ND	35.5
**Ulnar nerve**			
MCV (m/s)	>47	ND	61.3
Distal CMAP (mV)	>3	ND	6.6
Proximal CMAP (mV)		ND	5.9
DML (ms)	<4	ND	2.8
F wave occurrence (%)	>70	ND	100.0
Minimum F wave latency (ms)	<27	ND	24.9
SCV (m/s)	>46	ND	55.3
SNAP (μV)	>9	ND	33.0
**Tibial nerve**			
MCV (m/s)	>39	47.7	47.9
Distal CMAP (mV)	>3	7.6	6.5
Proximal CMAP (mV)		6.8	5.5
DML (ms)	<6.3	3.8	3.2
F wave occurrence (%)	>70	100.0	100.0
Minimum F wave latency (ms)	<58	48.1	48.8
**Sural nerve**			
SCV (m/s)	>39	58.7	61.4
SNAP (μV)	>4	10.0	11.1

*MCV, motor conduction velocity; CMAP, compound muscle action potential; DML, distal motor latency; SCV, sensory conduction velocity; SNAP, sensory nerve action potential; ND, not done*.

**Figure 3:**
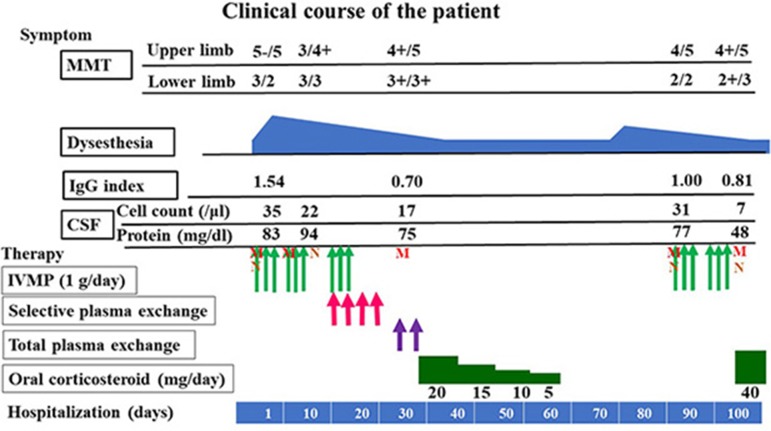
Clinical course and IgG index. MMT, manual muscle testing; CSF, cerebrospinal fluid; IVMP, intravenous methylprednisolone pulse therapy; M, magnetic resonance imaging; N, Nerve conduction study.

After the first total plasma exchange (1.25 plasma volume exchange with fresh frozen plasma replacement fluid; total plasma exchange procedures were performed twice a week for 1 week), muscle strength in the right upper limb and lower extremities improved and the patient could walk with a walker on day 28 post-admission. CSF analysis revealed improved cell count (17 cells/μL), total protein levels (75 mg/dL), and IgG index (0.70). Because MRI showed that abnormal enhanced lesions remained in the medullary cone and cauda equina (Figure 1), the patient started a regimen of oral prednisolone (PSL; 20 mg/day; 0.5 mg/kg/day) after the second plasma exchange, on day 35 post-admission, and she was discharged on day 40 post-admission (Figure 3). Her daily dose of PSL was tapered off, decreasing by 5 mg/week, and was finally suspended on day 68 post-admission

**Figure 4:**
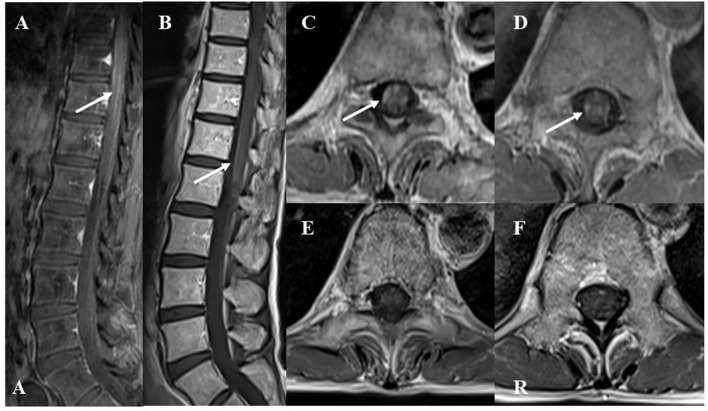
Gadolinium-enhanced spinal T1-weighted magnetic resonance imaging (MRI) findings at recurrence. The lower “A” represents “anterior” in **(A)**. The “R” represents “right” in **(F)**. **(A)** Sagittal MRI showed abnormal enhanced lesions in the epiconus to cauda equina. **(B)** After two courses of high-dose steroid pulse therapy, follow-up MRI showed decreased enhancement in the epiconus to cauda equina at recurrence. **(C,D)** Axial images showed abnormal enhanced lesions at the 9 and 12th levels of the thoracic spine. **(E,F)** After two courses of high-dose steroid pulse therapy, follow-up MRI showed decreased enhancement at the 9 and 12th levels of the thoracic spine.

After PSL was discontinued, the patient had progressive muscle weakness in the lower limbs and was readmitted at day 51 post-discharge. At the time of readmission, she needed to use a wheelchair. NCS revealed prolonged DML and decreased motor nerve conduction velocity (MCV) in the right tibial and peroneal nerves (DML of right tibial nerve and right peroneal nerve: 6.8 and 6.6 ms, respectively; MCV of right tibial nerve and right peroneal nerve: 32 and 35 m/s, respectively; Figure 2). MCV was also decreased in the left tibial nerve (36 m/s). Amplitudes of sensory nerve action potentials were not recordable in the bilateral sural nerves (Table 4). CSF analysis showed elevated cell counts (31 cells/μL) and total protein levels (77 mg/dL). Gadolinium-enhanced MRI demonstrated multiple foci of abnormal signal intensity from the epiconus to the cauda equina (Figure 4).

**Table 4:** Nerve conduction studies on both sides at the time of relapse.

	**Normal value**	**Left side**	**Right side**
Age at examination		60	
**Median nerve**			
MCV (m/s)	>47	ND	53.5
Distal CMAP (mV)	>4	ND	13.0
Proximal CMAP (mV)		ND	8.7
DML (ms)	<4.5	ND	4.2
F wave occurrence (%)	>70	ND	ND
Minimum F wave latency (ms)	<31	ND	ND
SCV (m/s)	>46	ND	55.1
SNAP (μV)	>9	ND	14.3
**Ulnar nerve**			
MCV (m/s)	>47	ND	56.3
Distal CMAP (mV)	>3	ND	10.9
Proximal CMAP (mV)		ND	10.4
DML (ms)	<4	ND	2.8
F wave occurrence (%)	>70	ND	ND
Minimum F wave latency (ms)	<27	ND	ND
SCV (m/s)	>46	ND	68.2
SNAP (μV)	>9	ND	38.7
**Tibial nerve**			
MCV (m/s)	>39	**36.1**	**32.1**
Distal CMAP (mV)	>3	8.4	11.5
Proximal CMAP (mV)		8.3	10.0
DML (ms)	<6.3	4.4	**6.8**
F wave occurrence (%)	>70	ND	93.0
Minimum F wave latency (ms)	<58	ND	53.3
**Peroneal nerve**			
MCV (m/s)	>40	ND	35.1
Distal CMAP (mV)	>2.5	ND	**2.3**
Proximal CMAP (mV)		ND	2.5
DML (ms)	<5.5	ND	**6.6**
F wave occurrence (%)	>70	ND	ND
Minimum F wave latency (ms)	<56	ND	ND
**Sural nerve**			
SCV (m/s)	>39	**Not evoked**	**Not evoked**
SNAP (μV)	>4	**Not evoked**	**Not evoked**

*MCV, motor conduction velocity; CMAP, compound muscle action potential; DML, distal motor latency; SCV, sensory conduction velocity; SNAP, sensory nerve action potential; ND, not done. Abnormal values are expressed in bold*.

Following treatment with methylprednisolone pulse therapy (1,000 mg/day for 3 days, two times), muscle strength in both lower limbs improved, NCS was normalized (Table 5), and abnormal signal intensities were decreased on the follow-up MRI (Figures 2, 4). Oral PSL (40 mg/day; 1 mg/kg/day) was initiated after high-dose methylprednisolone pulse therapy (Figure 3). Because she could walk with a walker and her CSF analyses were improved, she was discharged on day 19 post-readmission.

**Table 5:** Nerve conduction studies on both sides after two courses of high-dose steroid pulse therapy at the time of relapse.

	**Normal value**	**Left side**	**Right side**
Age at examination		60	
**Median nerve**			
MCV (m/s)	>47	53.7	57.2
Distal CMAP (mV)	>4	10.0	4.2
Proximal CMAP (mV)		9.2	4.1
DML (ms)	<4.5	3.0	3.2
F wave occurrence (%)	>70	93.0	100.0
Minimum F wave latency (ms)	<31	26.1	24.4
SCV (m/s)	>46	58.4	57.3
SNAP (μV)	>9	55.7	61.9
**Ulnar nerve**			
MCV (m/s)	>47	58.1	60.6
Distal CMAP (mV)	>3	9.7	6.6
Proximal CMAP (mV)		8.7	5.6
DML (ms)	<4	2.5	2.4
F wave occurrence (%)	>70	100.0	100.0
Minimum F wave latency (ms)	<27	25.7	23.8
SCV (m/s)	>46	55.1	57.5
SNAP (μV)	>9	61.0	69.5
**Tibial nerve**			
MCV (m/s)	>39	42.3	42.5
Distal CMAP (mV)	>3	4.7	6.0
Proximal CMAP (mV)		4.7	5.0
DML (ms)	<6.3	3.6	3.2
F wave occurrence (%)	>70	100.0	ND
Minimum F wave latency (ms)	<58	47.8	ND
**Sural nerve**			
SCV (m/s)	>39	46.4	48.6
SNAP (μV)	>4	10.2	12.4

*MCV, motor conduction velocity; CMAP, compound muscle action potential; DML, distal motor latency; SCV, sensory conduction velocity; SNAP, sensory nerve action potential; ND, not done*.

The daily dose of PSL was tapered off to 20 mg/day (0.5 mg/kg/day) by decreasing the dosage by 5 mg/month. At the time of publication, the patient remains in remission and NCS results (in both median, ulnar, tibial, peroneal, and sural nerves) have remained normal with the aid of low-dose PSL treatment (20 mg/day; 0.5 mg/kg/day) for 7 months so far (Table 6).

**Table 6:** Nerve conduction studies on both sides at 7 months after second admission.

	**Normal value**	**Left side**	**Right side**
Age at examination		60	
**Median nerve**			
MCV (m/s)	>47	ND	58.3
Distal CMAP (mV)	>4	ND	6.5
Proximal CMAP (mV)		ND	6.2
DML (ms)	<4.5	ND	3.2
F wave occurrence (%)	>70	ND	100.0
Minimum F wave latency (ms)	<31	ND	23
SCV (m/s)	>46	ND	57.6
SNAP (μV)	>9	ND	55.9
**Ulnar nerve**			
MCV (m/s)	>47	ND	60.2
Distal CMAP (mV)	>3	ND	8.9
Proximal CMAP (mV)		ND	7.8
DML (ms)	<4	ND	2.5
F wave occurrence (%)	>70	ND	100.0
Minimum F wave latency (ms)	<27	ND	23.6
SCV (m/s)	>46	ND	68.4
SNAP (μV)	>9	ND	31.8
**Tibial nerve**			
MCV (m/s)	>39	42.4	39.4
Distal CMAP (mV)	>3	10.4	7.0
Proximal CMAP (mV)		7.2	6.0
DML (ms)	<6.3	4.7	4.0
F wave occurrence (%)	>70	100.0	100.0
Minimum F wave latency (ms)	<58	46.6	48.0
**Peroneal nerve**			
MCV (m/s)	>40	43.9	43.2
Distal CMAP (mV)	>2.5	**1.3**	**2.2**
Proximal CMAP (mV)		**1.1**	**1.8**
DML (ms)	<5.5	4.5	5.4
F wave occurrence (%)	>70	93.0	93.0
Minimum F wave latency (ms)	<56	49.2	48.7
**Sural nerve**			
SCV (m/s)	>39	ND	46.6
SNAP (μV)	>4	ND	12.4

*MCV, motor conduction velocity; CMAP, compound muscle action potential; DML, distal motor latency; SCV, sensory conduction velocity; SNAP, sensory nerve action potential; ND, not done. Abnormal values are expressed in bold*.

## Discussion

We diagnosed our case with CCPD based on the diagnostic criteria of Ogata et al. (2). To date, there have been few reports of CCPD with RCF in NCS examination, including in patients positive for anti-galactocerebroside or -lactosylceramide antibody (1, 4, 5, 11); however, no study has focused on RCF in CCPD patients. RCF is characterized by a rapidly reversible nerve conduction blockade or slowing (12). This phenomenon is often observed in patients with axonal Guillain-Barré syndrome, with specific antibodies including anti-GM1 and -GD1a antibodies. These antibodies cause physiological conduction blocking at the axolemma at nodes of Ranvier. Some damaged nerve fibers can rapidly recover without remyelination. This reversible functional impairment results in RCF (12–14). In our patient with CCPD, anti-lactosylceramide antibody was positive, and RCF was observed twice. The RCF seen in the present case suggested axonal neuropathy.

We believe our case is CCPD. SSEP were reduced in amplitude, and NCS revealed a reduction in amplitude and the absence of an F wave in both peroneal nerves. RCF is often observed in axonal neuropathy. These findings therefore indicate that our case is demyelinating with axonal disorder, just like EMRN. Our case is considered unique in that it exhibited some common features of EMRN such as axonal demyelinating neuropathy, RCF, some dysautonomia, high levels of myelin basic protein, and positive OCB (5, 10, 11). Compared with previous patients with EMRN, our case had a relapsing–remitting clinical course (11). Because of the short-term follow-up periods, few studies have reported the recurrence of EMRN (11). Nanaura et al. were the first to report an anti-lactosylceramide-antibody-positive EMRN patient who had a relapsing–remitting clinical course and RCF (11). It may be possible that CCPD with RCF can occur in the early stages of EMRN.

In our case, peripheral nervous system lesions showed a good response to methylprednisolone, but CNS lesions had a poor response to methylprednisolone, as previously reported (4, 5, 11). RCF in our patient suggested that anti-lactosylceramide antibody caused physiological reversible conduction blocking in the peripheral nervous system, similar to that of axonal Guillain-Barré patients (12–14). Abundant lactosylceramide and anti-lactosylceramide antibodies in the CNS may induce superoxide generation from neutrophils and lead to inflammation in the CNS (6, 8, 9). Repulsive guidance molecule A and semaphorin 3A inhibit axonal growth in CNS lesions (15, 16). Therefore, abundant lactosylceramide, repulsive guidance molecule A, and semaphorin 3A might disturb the recovery of CNS lesions, although more work is needed in the future to verify this possibility.

Our patient required total plasma exchange in addition to selective plasma exchange at the initial attack only, and there was a correlation between clinical symptoms and the IgG index (Figure 4). This finding indicates that the reduction of antibody titers of IgG is important and that inflammation in the CNS was also induced by other more giant molecules and cells, such as IgM, cytokines, neutrophils, and T cells in the acute phase (17). Therefore, in the future we should perform total plasma exchange for anti-lactosylceramide-antibody-positive CCPD with RCF or EMRN patients whose symptoms do not respond sufficiently to immunotherapy or selective plasma exchange, especially in the early stages.

In conclusion, clinicians need to examine serum and CSF anti-lactosylceramide antibodies in CCPD patients with RCF. Re-examination of NCS may be important to discriminate between CCPD and CCPD with RCF (EMRN). Total plasma exchange was effective in the acute phase, and oral corticosteroid treatment was effective for relapse prevention.

## Data Availability

All datasets generated for this study are included in the manuscript and/or the supplementary files.

## Ethics Statement

No investigations or interventions were performed outside of routine clinical care for this patient. As this is a case report, without experimental intervention into routine care, no formal research ethics approval was required. Written, fully informed consent for the publication of this case report and participation in the study was obtained from the patient. This case study describes routine clinical care provided for a patient only.

## Author Contributions

MH and SM were responsible for the overall study design and wrote the manuscript. YU, SS, and TMu contributed to data acquisition. TMo, TK, KI, and TH contributed to the analysis and interpretation of the data. SM, HK, TMu and TT contributed to the drafting and critical revision of parts of the submitted materials.

### Conflict of Interest Statement

The authors declare that the research was conducted in the absence of any commercial or financial relationships that could be construed as a potential conflict of interest.
